# Spontaneous Hemodynamic Oscillations during Human Sleep and Sleep Stage Transitions Characterized with Near-Infrared Spectroscopy

**DOI:** 10.1371/journal.pone.0025415

**Published:** 2011-10-17

**Authors:** Tiina Näsi, Jaakko Virtanen, Tommi Noponen, Jussi Toppila, Tapani Salmi, Risto J. Ilmoniemi

**Affiliations:** 1 Department of Biomedical Engineering and Computational Science (BECS), Aalto University, Aalto, Espoo, Finland; 2 BioMag Laboratory, HUSLAB, Helsinki University Central Hospital, Helsinki, Finland; 3 Department of Nuclear Medicine and Turku PET Centre, Turku University Hospital, Turku, Finland; 4 Department of Clinical Neurophysiology, Helsinki University Central Hospital, Helsinki, Finland; Cuban Neuroscience Center, Cuba

## Abstract

Understanding the interaction between the nervous system and cerebral vasculature is fundamental to forming a complete picture of the neurophysiology of sleep and its role in maintaining physiological homeostasis. However, the intrinsic hemodynamics of slow-wave sleep (SWS) are still poorly known. We carried out 30 all-night sleep measurements with combined near-infrared spectroscopy (NIRS) and polysomnography to investigate spontaneous hemodynamic behavior in SWS compared to light (LS) and rapid-eye-movement sleep (REM). In particular, we concentrated on slow oscillations (3–150 mHz) in oxy- and deoxyhemoglobin concentrations, heart rate, arterial oxygen saturation, and the pulsation amplitude of the photoplethysmographic signal. We also analyzed the behavior of these variables during sleep stage transitions. The results indicate that slow spontaneous cortical and systemic hemodynamic activity is reduced in SWS compared to LS, REM, and wakefulness. This behavior may be explained by neuronal synchronization observed in electrophysiological studies of SWS and a reduction in autonomic nervous system activity. Also, sleep stage transitions are asymmetric, so that the SWS-to-LS and LS-to-REM transitions, which are associated with an increase in the complexity of cortical electrophysiological activity, are characterized by more dramatic hemodynamic changes than the opposite transitions. Thus, it appears that while the onset of SWS and termination of REM occur only as gradual processes over time, the termination of SWS and onset of REM may be triggered more abruptly by a particular physiological event or condition. The results suggest that scalp hemodynamic changes should be considered alongside cortical hemodynamic changes in NIRS sleep studies to assess the interaction between the autonomic and central nervous systems.

## Introduction

Sleep is a part of constantly ongoing homeostatic regulation between neurons, glial cells, and vasculature of the brain to maintain health, adaptability, and cognitive performance [1]. Slow-wave sleep (SWS), also known as deep sleep, differs from the physiologically distinct light sleep (LS) and rapid-eye-movement sleep (REM) stages by a dramatic increase in synchronized neuronal activity and loss of information integration across cortical areas [2], [3]. This activity plays a crucial role in cortical plasticity and is seen in electroencephalographic (EEG) recordings as large-amplitude delta waves (0.5–2 Hz) [3], [4]. LS and particularly REM generally display much more complex and chaotic EEG patterns [2]. However, the brain also exhibits slow physiological oscillations which cannot be studied with EEG alone [5]. To form a comprehensive understanding of the neurophysiologic interactions related to sleep, bioelectric signals must be supplemented with hemodynamic and metabolic data [6]–[10].

Of the methods available for studying cerebral hemodynamics and metabolism, positron emission tomography (PET) and functional magnetic resonance imaging (fMRI) are poorly suited for all-night sleep measurements. Both methods require the subject to lie still, and in the case of PET also ionizing radiation and poor temporal resolution limit applicability. Transcranial Doppler sonography (TCD) is particularly well suited for monitoring global cerebral blood flow (CBF), but it is insensitive to blood oxygenation changes and does not reveal regional CBF (rCBF). In contrast to the aforementioned methods, near-infrared spectroscopy (NIRS) tolerates small movements of the head, can measure cortical hemodynamic and oxygenation changes locally, and allows tracking the concentration changes of both oxygenated (Δ[HbO_2_]) and deoxygenated (Δ[HbR]) hemoglobin (Hb). These features, combined with comfortability, portability, and relatively low cost of equipment, make NIRS an excellent method for monitoring hemodynamics during sleep [11]–[14].

Spontaneous hemodynamic activity can be grouped into high-frequency (HFO, 150–400 mHz), low-frequency (LFO, 40–150 mHz), and very-low-frequency oscillations (VLFO, 3–40 mHz) [15]. This division is to some extent arbitrary, and the exact frequency bands vary between authors [16]. HFOs in NIRS signals are typically attributed to respiration [16]–[18]. LFOs and VLFOs can be traced to multiple underlying factors, including oscillating neuronal activity [5], [19]–[22], vasomotion [17], [23], [24], and autonomic control of heart rate (HR) and blood pressure (BP) [18]. They are influenced by numerous independent factors, such as age, physical activity, mental and physical stress, task conditions, and the circadian cycle [5], [25].

NIRS sleep studies have primarily concentrated on investigating sleep stage transitions, often during daytime napping [10], [26], baseline changes in hemodynamic parameters between sleep stages [11], [27]–[29], or the effects of sleep-disordered breathing on cerebral hemodynamics [14], [30]. Almost no attention has been paid to transitions related to SWS [27], and the behavior of LFOs and VLFOs in different sleep stages have not been studied previously with NIRS.

In the present study, we examine spontaneous hemodynamic oscillations measured with NIRS in different sleep stages. We also investigate hemodynamic changes during transitions between sleep stages, particularly transitions to and from SWS, and contrast cortical, scalp, and systemic hemodynamic parameters with each other to determine how they are interrelated.

## Materials and Methods

### Participants

All-night NIRS–EEG sleep data were gathered from 13 healthy volunteers (9 males, 4 females, mean age 26 years, range 21–32). Each subject was measured during at least two nights to reduce the influence of the unfamiliar surroundings on sleep quality; however, no such influence was observed when comparing sleep time to total measurement time on the first and second measurement night. A total of 30 all-night measurements were conducted, with five measurements discarded due to poor quality of either NIRS or EEG data.

### Ethics

All subjects gave their written informed consent prior to the measurements. The study was approved by the Ethics Committee of Helsinki University Central Hospital and was in compliance with the Finnish legislation and the declaration of Helsinki.

### Data Acquisition

**Figure 1:**
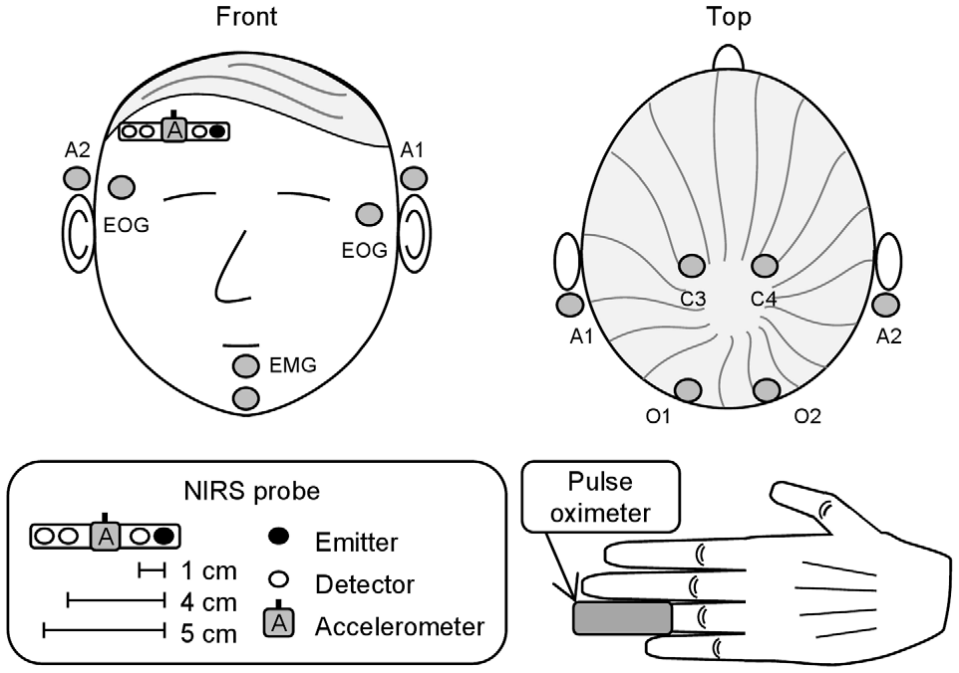
Measurement setup. The placement of the NIRS probe, accelerometer, pulse oximeter, and polysomnography electrodes.

The NIRS data were recorded with a frequency domain instrument using 685-nm and 830-nm light [31]. An optical probe with a single light emitter fiber and three detector fiber bundles was placed on the right side of the subject's forehead just below the hairline, above the right prefrontal cortex (Fig. 1). The emitter–detector separations were 1, 4, and 5 cm, with the 1-cm channel measuring mainly scalp hemodynamics and the 4- and 5-cm channels probing also cortical hemodynamics [32]. Light attenuation changes in tissue were converted into Δ[HbO_2_] and Δ[HbR] with the modified Beer–Lambert law (MBLL) [33]. The sum Δ[HbO_2_]+Δ[HbR] is an indicator of blood volume changes, whereas blood flow changes cannot be directly inferred from NIRS signals. However, due to the passive nature of blood transport from the brain, cerebral blood volume (CBV) changes are often indicative of parallel CBF changes [34].

MBLL requires setting the photon pathlength in tissue and the specific extinction coefficients of HbR and HbO_2_. Although the frequency domain technique allows estimating the pathlength separately for each individual [35], technical difficulties prevented this in ten measurements. Thus, an average differential path length factor was estimated from the available phase data (20 measurements) and used for calculating the photon pathlength for all subjects. The estimated differential path length factor was 6.16 for 685 nm and 5.84 for 830 nm. Values for the specific extinction coefficients of HbR and HbO_2_ were taken from literature [36].

An accelerometer attached to the NIRS probe recorded head movements that might disturb the tissue–optode contact and thus introduce motion artifacts to the data (Fig. 1). An automated algorithm was used to identify and correct motion artifacts that appear as large discontinuities in the Δ[HbO_2_] and Δ[HbR] baselines [37]. These discontinuities were removed on the assumption that actual physiological concentration baseline changes during brief movements are negligible. This correction allowed tracking concentration changes over sleep stage transitions involving motion.

Δ[HbO_2_] and Δ[HbR] were estimated at a sampling rate of approximately 10 Hz and resampled to a uniform rate of exactly 10 Hz with spline interpolation. Oscillations related to respiration and heart beat were removed by low-pass filtering the concentration signals (type II Chebyshev filter, passband edge at 150 mHz). The 1-cm channel was selected to represent extracerebral hemodynamics and the 4-cm channel to represent cortical hemodynamics, since it had a better signal-to-noise ratio than the 5-cm channel. Otherwise, the 4-cm and 5-cm signals behaved similarly.

An all-night polysomnogram consisting of EEG (channels C4-A1, O2-A1, C3-A2, and O1-A2), left and right electrooculograms (EOG), and a chin electromyogram (EMG) was recorded from all subjects (Fig. 1). Based on the polysomnogram, a neurophysiologist scored the sleep into S1, S2, S3, S4, REM, wake (W), and movement time stages in 30-s epochs according to the Rechtschaffen–Kales rules [38]. For data analysis, the S3 and S4 non-REM (NREM) stages were combined into SWS, and the S1 and S2 NREM stages into LS. Also, if two similarly scored 30-s epochs were separated by one 30-s epoch scored differently, that epoch was rescored to match its surroundings. This classification corresponds closely to the new sleep scoring guidelines given in the American Academy of Sleep Medicine scoring manual [39], and clearly distinguishes fundamentally different EEG states from each other [2]. It also produces longer homogeneously scored periods, facilitating the analysis of VLFOs.

A finger pulse oximeter measured the photoplethysmographic waveform and the 10-s average of the peripheral arterial oxygen saturation (SpO_2_). The peak-to-peak pulsation amplitude of the photoplethysmographic waveform (PPGamp) and HR were derived from the pulse oximeter signal and interpolated to a uniform sampling rate of 1 Hz and filtered similarly to the NIRS signals (type II Chebyshev filter, passband edge at 150 mHz). PPGamp reflects the amount of blood pulsating in the blood vessels of the finger and normally decreases with vasoconstriction and increases with vasodilation [40]. PPGamp is also negatively correlated with systolic BP in normal sleep, although this correlation decreases in REM sleep [41].

### Data Analysis

#### Spontaneous hemodynamic oscillations

For each continuous period of SWS, LS, REM, and W, Δ[HbO_2_] and Δ[HbR] signal power in the VLFO and LFO bands was estimated with Welch's power spectral density (PSD) method with 6.8-min segment length (2^12^ samples of NIRS data), 50-% segment overlap, and 1.2-mHz frequency resolution. Only sleep periods lasting at least 13.7 min (2^13^ samples of NIRS data) were included, so that at least three segments were averaged for each PSD (Table 1). Logarithmic signal powers were tested for statistically significant (*p*<0.05) differences between sleep stages with one-way ANOVA followed by Tukey's honestly significant difference (HSD) *post-hoc* test. Separate tests were conducted for each frequency band, NIRS channel, and Hb species. To account for multiple comparisons, the ANOVA *p*-values were controlled with the false discovery rate (FDR) method [42]. The PSDs of HR, PPGamp, and SpO_2_ were analyzed similarly, except the LFO band for SpO_2_ was restricted to 40–50 mHz because of the 100-mHz sampling rate, and multiple comparisons were controlled for only over the two frequency bands.

**Table 1:** Sleep periods (number/duration in min) included in the PSD analysis.

Subject, gender	SWS	LS	REM	W	Total
1, M	8/147.5	30/635.5	13/324.5	1/19	**52/1126.5**
2, M	3/55	11/259	4/101.5	0/0	**14/415.5**
3, M	4/129	10/249	5/137.5	2/194	**21/709.5**
4, M	2/66	7/170	1/17	2/51.5	**12/304.5**
5, M	4/106	13/300	4/95	2/87	**23/588**
6, F	3/57.5	17/477.5	6/99.5	3/54	**29/688.5**
7, M	1/16.5	19/478.5	2/37.5	0/0	**22/532.5**
8, M	1/49	5/115.5	0/0	2/224.5	**8/389**
9, F	4/105.5	13/350.5	3/119.5	3/202.5	**23/778**
10, F	1/15	10/594	3/69.5	0/0	**14/678.5**
11, M	3/96	5/104.5	1/24	8/418.5	**17/643**
12, F*	N/A	N/A	N/A	N/A	**N/A**
13, M	5/136	14/345	4/109.5	1/14.5	**24/605**
**Total**	**39/9790 min**	**154/4079 min**	**46/1135 min**	**24/1265.5 min**	**263/7458.5 min**

• Both measurements rejected due to poor data quality.

#### Sleep stage transitions

Sleep stage transitions were analyzed for data segments where the sleep stages preceding and following the transition were maintained for at least 300 s. Transitions not involving LS were omitted due to their rarity. For each transition, the means of Δ[HbO_2_], Δ[HbR], HR, PPGamp, and SpO_2_ for the 300 s preceding the transition were set to zero. Then, time courses of these five parameters were averaged for each of the six transition types (SWS→LS, LS→SWS, LS→REM, REM→LS, W→LS, and LS→W).

In addition to the average time courses, transition-related baseline changes in all five parameters were investigated. The mean value of each parameter between 300 and 200 s before the transition was subtracted from the mean between 200 and 300 s after the transition for each individual transition recorded. These baseline differences were tested for statistically significant deviation from zero with Student's *t*-test for each transition type separately. For Δ[HbO_2_] and Δ[HbR], the significance level (*p*<0.05) was adjusted with the FDR method for multiple comparisons from the two Hb species, six transition types and two channels (1 and 4 cm). For HR, PPGamp, and SpO_2_, the FDR adjustment was done to account for the six transition types.

The contribution of extracerebral hemodynamics to the 4-cm signals was evaluated with principal component analysis (PCA) of the 1- and 4-cm average time courses. Briefly, the method separates statistically uncorrelated components from NIRS data, so that it allows identifying hemodynamic changes that are present in the brain but not in the scalp [43]. Δ[HbO_2_] and Δ[HbR] were analyzed separately. The PCA component with the highest contribution to the 1-cm signal was removed from the 4-cm signal, and the remaining 4-cm Δ[HbO_2_] and Δ[HbR] were evaluated visually. PCA was also extended to include HR, PPGamp, and SpO_2_ to investigate their similarity to cortical hemodynamics. This analysis was done by first removing a component corresponding to one of the systemic variables from the 1- and 4-cm signals, and then removing the 1-cm component from the 4-cm signal.

Spontaneous hemodynamic oscillations before and after each transition were quantified by calculating the standard deviation (SD) of Δ[HbO_2_], Δ[HbR], HR, PPGamp, and SpO_2_ over six non-overlapping 100-s segments (−300…−200 s, −200…−100 s, etc., with sleep stage transition at 0 s). The SD data from individual transitions were then averaged to arrive at an average time course representing combined VLFO and LFO amplitude changes before and after the transition. The first and last segments were compared for statistically significant differences with Student's *t*-test with the FDR adjustment.

All signal processing was carried out in MATLAB (The MathWorks, Inc., Natick, Massachusetts, USA). Data segments clearly containing large motion or signal processing artifacts were not analyzed.

## Results

### Spontaneous hemodynamic oscillations

**Figure 2:**
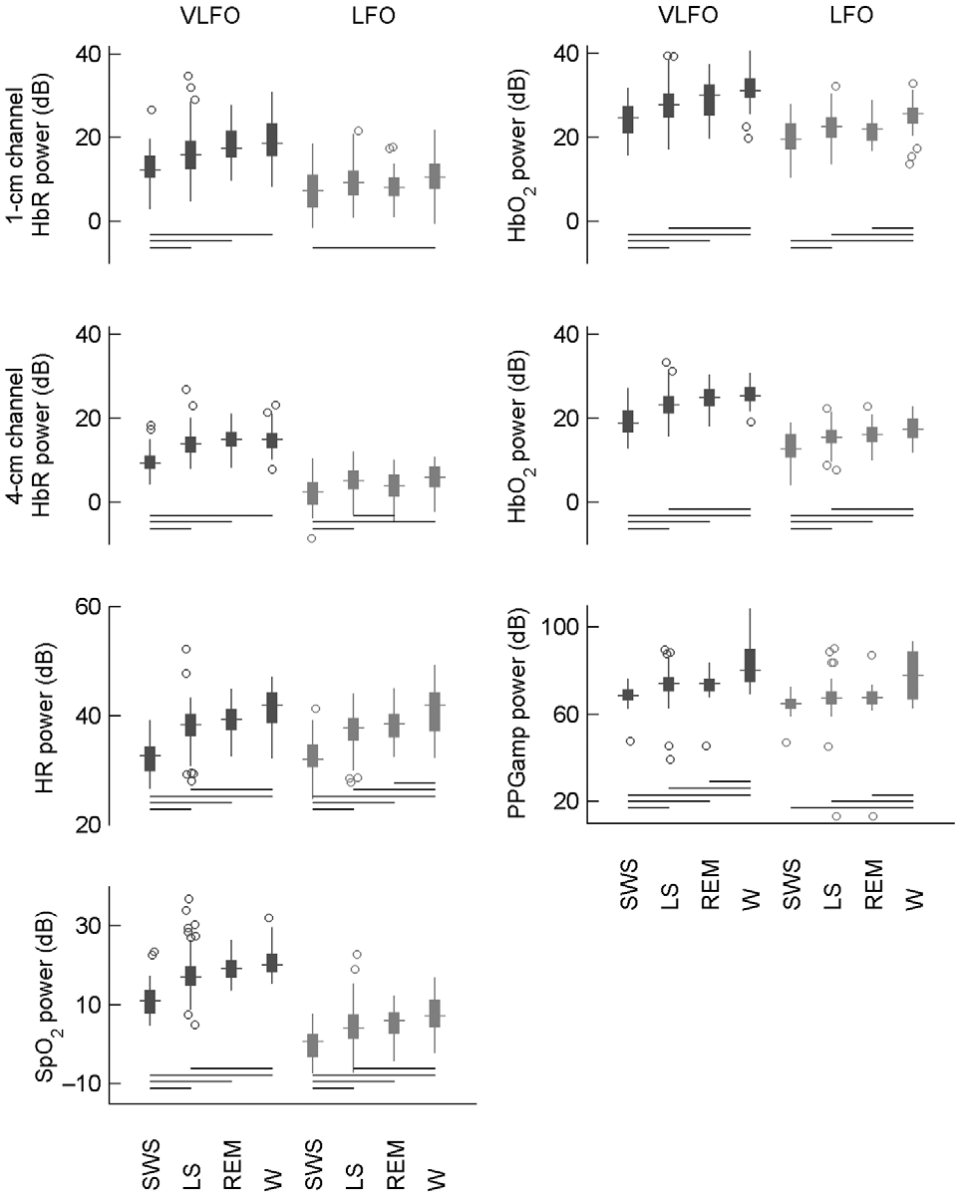
Logarithm of signal power in the VLFO and LFO bands in different sleep stages. For each stage, the box plot gives the distribution of power for NIRS and systemic data in the sleep periods of Table 1. The box corresponds to the interquartile range (IQR) of the distribution, and the horizontal line crossing the box marks the median. The whiskers indicate values that fall within 1.5 IQR of the box, and any remaining outliers are marked with circles. The horizontal lines show which sleep stages differ from each other statistically significantly (one-way ANOVA corrected with FDR and Tukey's HSD criterion, *p*<0.05). VLFO and LFO power levels are presented on the same scale for convenience, but they are not directly comparable with each other due to 1/*f* noise in the PSDs [44]. Note that the oscillatory powers are smaller in SWS compared to other sleep stages and typically increase in the order SWS–LS–REM–W.

Spectral analysis of the NIRS and systemic signals showed that the VLFO and LFO powers in SWS mostly differed statistically significantly from the powers in the other stages, particularly in the VLFO band (Fig. 2). In both bands, the power level increased typically in the order SWS–LS–REM–W, although the differences between LS and REM were mostly non-significant. Oscillation power was also higher in Δ[HbO_2_] than in Δ[HbR].

**Figure 3:**
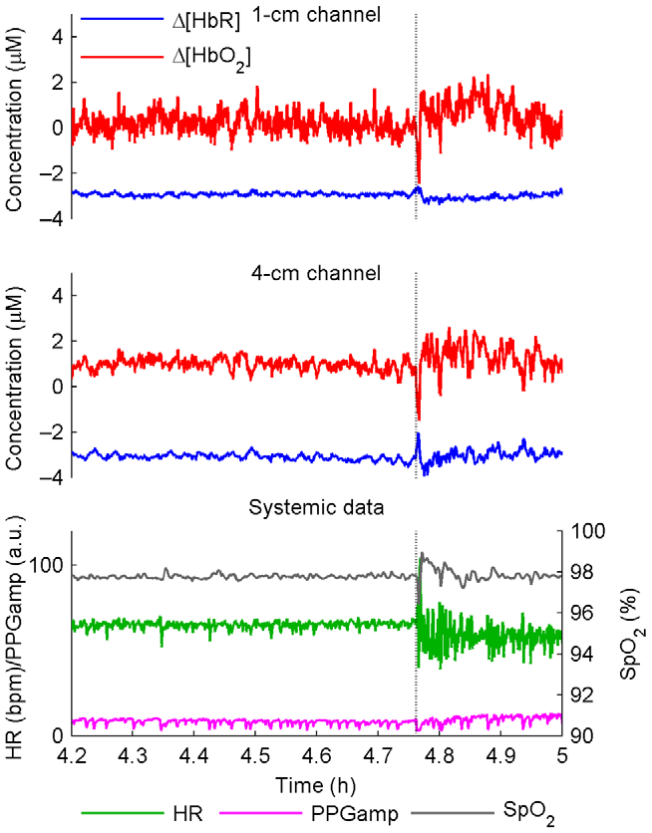
Time-domain behavior of signals in sleep in a typical subject. The vertical dotted line indicates a SWS→LS transition. Spectral content above 150 mHz has been removed with low-pass filtering. The concentration baselines have been chosen arbitrarily to avoid overlapping of the curves. Note the stability of the hemodynamic oscillations in SWS and the increase in hemodynamic activity after the transition to LS.

Figure 3 shows the evolution of hemodynamic signals in the VLFO and LFO bands in the SWS→LS transition for a typical subject. The signals were relatively stable in SWS, with hemodynamic activity clearly increasing after the transition to LS, particularly in the 4-cm detector. This, along with similar time and frequency domain results from other subjects, indicates suppression of hemodynamic oscillations during SWS.

### Sleep stage transitions

**Figure 4:**
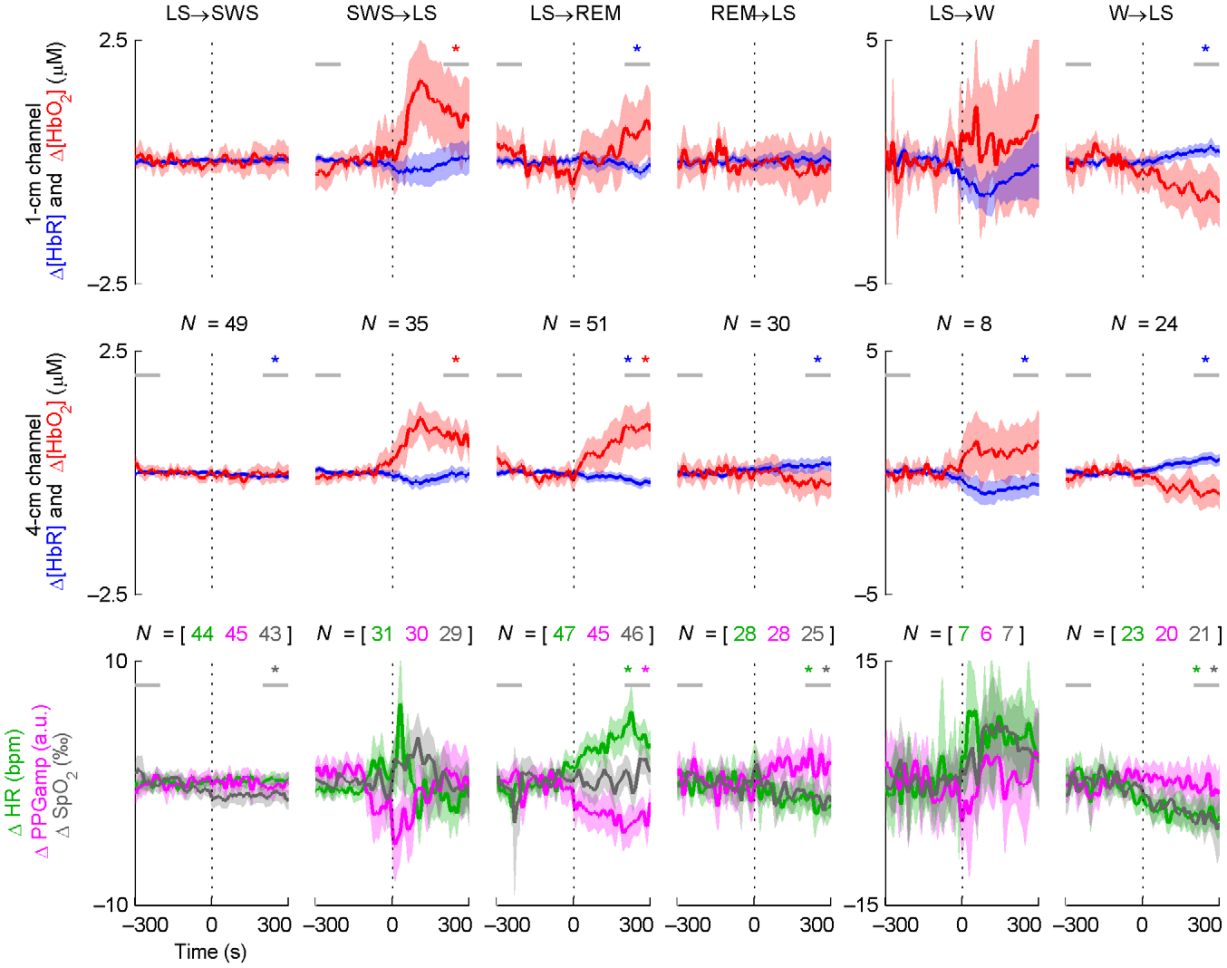
Time course averages around sleep stage transitions. *N* indicates the number of transitions (vertical dotted line) averaged and is equal for the two NIRS channels. The shading indicates the 95-% confidence interval of the mean. Statistically significant baseline changes are indicated with a star of the corresponding color; the horizontal grey lines indicate the 100-s periods the baseline comparison was based on. The results from the baseline comparisons are not directly comparable to the confidence intervals, since paired *t*-tests were used for the former. For graphical purposes, LFOs were removed from the signals by low-pass filtering (passband edge at 40 mHz). This did not affect the statistical tests or the overall behavior of the curves. Note the three different transition types (persistent changes, transient changes, or no changes at all) and the asymmetry of opposing transitions in terms of the magnitude and speed of changes (e.g. LS→SWS vs. SWS→LS). Δ[HbR] and PPGamp generally change in the opposite direction compared to Δ[HbO_2_] and HR.

Average Δ[HbR] and Δ[HbO_2_] time courses for the SWS→LS and LS→REM transitions indicated an increase in blood volume in the 1- and 4-cm NIRS channels (Fig. 4). These increases were accompanied by a simultaneous increase in HR and a decrease in PPGamp. The changes related to the SWS→LS transition appeared to be mostly transient, except for the increase in Δ[HbO_2_], while changes at the LS→REM transition were more persistent. The opposite transitions, LS→SWS and REM→LS, were involved with smaller and opposite changes, or no changes at all. The W→LS transition was accompanied by an increase in Δ[HbR] and decreases in Δ[HbO_2_], HR, and SpO_2_, while opposite changes arose in the LS→W transition. In the latter case, the changes were mostly not statistically significant due to the small number of transitions recorded.

Removing extracerebral contribution with PCA affected the interpretation of the 4-cm signals mainly in the SWS→LS transition by removing the prominent transient changes (Fig. S1). However, when components corresponding to both HR and the 1-cm signal were removed with PCA, no apparent transition-related responses were present in any of the remaining 4-cm signals (Fig. S1). Removing components corresponding to PPGamp and SpO_2_ did not have a similar effect (not shown).

**Figure 5:**
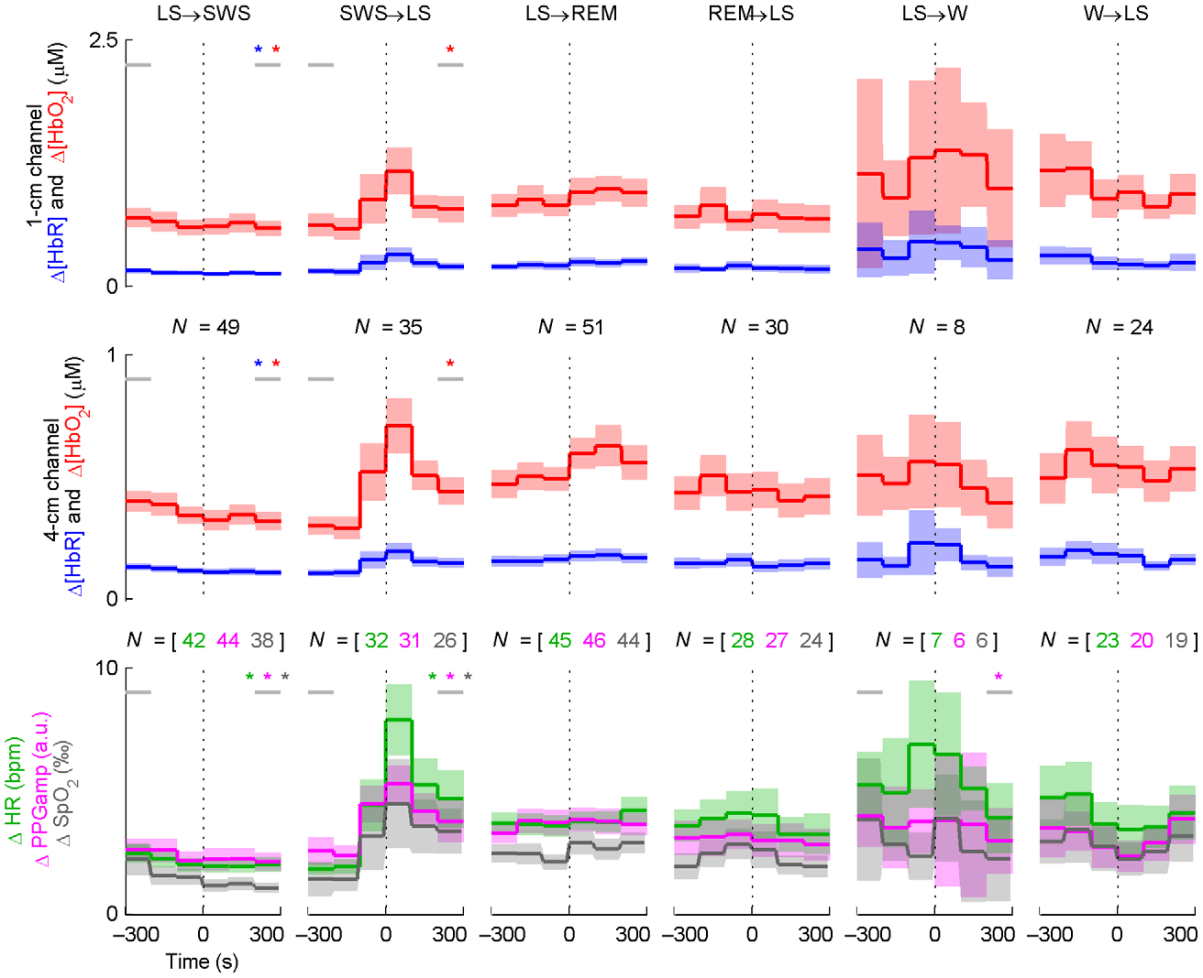
SDs of hemodynamic parameters over 100-s segments in sleep stage transitions. Statistically significant changes in SDs are indicated with a star of the corresponding color and 95-% confidence intervals with shading as in Figure 4. In NIRS signals, the only statistically significant transitions are the ones involving SWS, demonstrating the lower level of spontaneous hemodynamic activity in SWS.

SD changes in sleep stage transitions indicated differences in spontaneous hemodynamic activity primarily between SWS and LS (Fig. 5). This difference was clearly visible in both LS→SWS and SWS→LS transitions. In the LS→SWS transition, the level of spontaneous activity appeared to change gradually, whereas in the SWS→LS transition the transient changes visible in the average time courses (Fig. 4) dominate. However, also in the SWS→LS transition the level of spontaneous activity appeared to be higher after the transient. The results are in line with the PSD analysis of different sleep stages where the power of VLFOs and LFOs decreased in SWS compared to other stages. No statistically significant SD differences between LS and W or LS and REM were observed in the NIRS signals.

## Discussion

Our investigation revealed two major findings. First, slow spontaneous hemodynamic activity was greatly reduced in SWS compared to other sleep stages, while differences between LS and REM were relatively small. The reduction occurred simultaneously with the transition to SWS and was reversed with the transitioning away from SWS. Second, opposite sleep stage transitions (e.g., LS→SWS and SWS→LS) exhibited asymmetric behavior of the hemodynamic variables in the time domain. Transitions associated with an increase in the complexity of cortical activity (SWS→LS, LS→REM, LS→W) were characterized by large, relatively fast transient or persistent hemodynamic changes (Δ[HbO_2_] and HR increases and Δ[HbR] and PPGamp decreases). Transitions involving a reduction in the complexity of cortical activity (LS→SWS, REM→LS, W→LS) led to opposite but small and gradual changes, or no changes at all.

### Reduction of spontaneous hemodynamic activity in SWS

The reduction in the slow hemodynamic oscillations in SWS coincides with the presence of delta waves in the EEG, suggesting a possible common origin for them. Since the delta waves are generated by neuronal activity that is relatively constant over the local cortical area [4], variations in rCBF could also be expected to be suppressed in SWS. Since suppression of hemodynamic oscillations was evident in both systemic and cortical signals during SWS, the question may also be raised whether there is a synergetic benefit from this simultaneous reduction. For example, since SWS has been associated with diminished vasoreactivity to hypoxia [45], this could necessitate the suppression of systemic hemodynamic oscillations to ensure constant CBF and cerebral oxygenation.

In contrast to our results on HR oscillations, in a previous study the relative contributions of VLFOs and LFOs in HR variability were observed to increase in REM sleep compared to NREM, while there were practically no such differences between LS and SWS [46]. However, the same study indicated that the absolute spectral power increases from SWS to LS to REM, which corresponds to our results. This latter pattern has also been observed for sympathetic nervous system activity, which plays a key role in the regulation of systemic hemodynamic variables and homeostasis [47].

### Sleep stage transitions

This is the first study to present a comprehensive account of the time evolution of hemodynamic parameters in sleep stage transitions. Our observations on the W→LS and LS→W transitions are consistent with a general reduction in physiological activity and alertness in sleep compared to wakefulness, and in agreement with previous studies [10], [11], [26]–[28]. In some cases, our results on sleep stage transitions cannot be directly compared with the results of previous studies since they have compared hemodynamics and metabolism between REM, SWS, and W instead of comparing these states to LS [27], [48]. In some studies, hemodynamic parameters such as rCBF have been studied as a function of EEG delta activity instead of sleep stage transitions [49]–[51].

In the SWS→LS transition, the systemic parameters were the first to react, suggesting peripheral vasoconstriction and an increase in HR. The sudden and transient nature of these changes suggests that the transition may be linked to a transient arousal [47]. The shift in systemic parameters is followed by NIRS signal changes indicating an increase in the cortical and scalp blood volumes. In contrast, TCD evidence suggests that arousals during NREM sleep not resulting in awakening are more likely to lead to a decrease in CBF and CBV [52]. We speculate that the hemodynamic changes at the SWS→LS transition may also play a significant role in the termination of SWS, e.g., by actively stimulating neurons into moving away from the delta wave activation pattern.

In the LS→REM transition, the changes in both systemic parameters and NIRS signals persisted well into the REM period, which may reflect the higher sympathetic activity in REM compared to other sleep stages [11], [47]. The onset of REM sleep is accompanied by rapid eye movements which have been reported to activate the prefrontal cortex in a previous NIRS study [11]. Thus, changes in cortical hemodynamics could be related to cerebral activity produced by dreaming. However, rapid-eye-movement-related fMRI studies have not shown activity in the prefrontal cortex close to our measurement site [53], [54].

A statistically significant change in the amplitude of VLFOs and LFOs was visible in all hemodynamic variables within 300 s of the LS→SWS transition, as well as in all variables except Δ[HbR] in conjunction with the opposite SWS→LS transition. The onset of SWS was primarily characterized by a reduction in the amplitude of slow hemodynamic oscillations, not as a baseline change in hemodynamic parameters. Since the oscillations are to at least some extent controlled by feedback loops in the autonomic nervous system, the results may be interpreted to reflect differences in homeostatic regulation between SWS and the other stages.

Many sleep disorders, such as obstructive sleep apnea and the restless legs syndrome, lead to an increase in the frequency of sleep stage transitions to LS and W, and a decrease in the duration of continuous periods spent in one stage [55], [56]. Since sleep stage transitions interfere with the prevalent homeostatic oscillation patterns, an increase in the transition frequency could contribute to the detrimental effects of sleep disorders by disrupting and damaging the homeostatic feedback system over the long term. Decreased cardiovascular oscillations have been associated with various diseases, including diabetes, obstructive sleep apnea, and cardiovascular mortality [57].

### Limitations

Our data are based on hemodynamic recordings from the right prefrontal cortex. While this is an inviting location to measure with NIRS during sleep due to the lack of hair and reduced risk of probe displacement from rubbing against the pillow, the results may not be applicable to other brain areas. Thus, optical topography would be necessary to compare hemodynamic signals between different cortical areas. Also, while we have presented and discussed here the general behavior of LFOs and VLFOs in different sleep stages, a more detailed analysis of specific frequency bands in the 0–150 mHz range would provide a more quantitative reference for future NIRS sleep studies.

We used a 4-cm NIRS channel to represent cortical hemodynamics. This carries the risk of attributing hemodynamic changes in the scalp to cortical circulation, especially since the 1- and 4-cm signals resemble each other to some extent. For example, the time domain behavior of the 4-cm NIRS signals in all sleep stage transitions could be explained by the influence of systemic hemodynamics, as was demonstrated by PCA with the 1-cm signal and HR as physiological noise models. However, this does not show that there are no cortical hemodynamic changes associated with the transitions, only that any such changes must correlate with systemic hemodynamic changes and cannot be isolated with PCA or any other method that relies on the systemic signals to represent noise. Moreover, since systemic hemodynamic changes reflect the behavior of the autonomic nervous system, they also reflect neurophysiological changes related to sleep. Since the influence of autonomic nervous system activity on cerebral hemodynamics is a relatively unknown subject [58], it is difficult to determine in what proportions the 4-cm signals represent contribution from surface tissue, cortical hemodynamic changes due to cortical activation, and cortical hemodynamic changes arising from systemic influences.

It is generally acknowledged that the baselines of hemodynamic parameters such as HR and BP change during the course of the night [46], [59]. In our data, instrumental drift prevented reliable tracking of Δ[HbO_2_] and Δ[HbR] baselines over several hours, and we did not compare VLFO and LFO power levels between different parts of the night. Investigating the overnight behavior of the parameters discussed in this study might also provide new insights into cerebral hemodynamics during sleep.

### Conclusions

This is the first effort to compare spontaneous hemodynamic VLFOs and LFOs between different sleep stages, and to present a comprehensive account of hemodynamic changes in sleep stage transitions to and from SWS. The results show that SWS is associated with a significantly lower level of slow spontaneous hemodynamic activity than LS, REM, and W. This may be explained by the differences in neuronal activation patterns between sleep stages as well as a reduction in autonomic nervous system activity. Also, transitions between sleep stages are asymmetric, so that the SWS→LS and LS→REM transitions corresponding to increasing complexity of neuronal activity are involved with more dramatic hemodynamic changes than the gradually occurring opposite transitions. This may be partly explained by increased sympathetic activity related to the transition or the new sleep stage. These observations indicate that monitoring spontaneous cerebral hemodynamic oscillations with NIRS provides novel information on the neurophysiological characteristics of sleep. They also encourage examining the interaction between autonomic and cortical hemodynamics in more detail.

## Supporting Information

Figure S1 **Time course averages around sleep transitions after PCA filtering.** The first row shows the original 4-cm transition averages, the second row the 4-cm averages after removing the component corresponding to the 1-cm averages, and the third row the 4-cm averages after removing components corresponding to HR and the 1-cm averages. LFOs have been removed from the signals for graphical purposes. Note that the PCA filtering removes all visible changes from the 4-cm averages, suggesting that any cortical hemodynamic changes either correlate with systemic hemodynamics or are too small to be detected. In some cases, the PCA-filtered averages of the second row still visually resemble the 1-cm averages. This is most likely because the identification and removal of the 1-cm component was based on a simple statistical criterion instead of visual inspection or a complex physiological or anatomical model.
(TIF)
