# Supplementary figures and images for: Spontaneous Hemodynamic Oscillations during Human Sleep and Sleep Stage Transitions Characterized with Near-Infrared Spectroscopy
Source: PLoS One. 2011 Oct 17;6(10):e25415. doi: 10.1371/journal.pone.0025415 (PMC3197192; doi:10.1371/journal.pone.0025415)

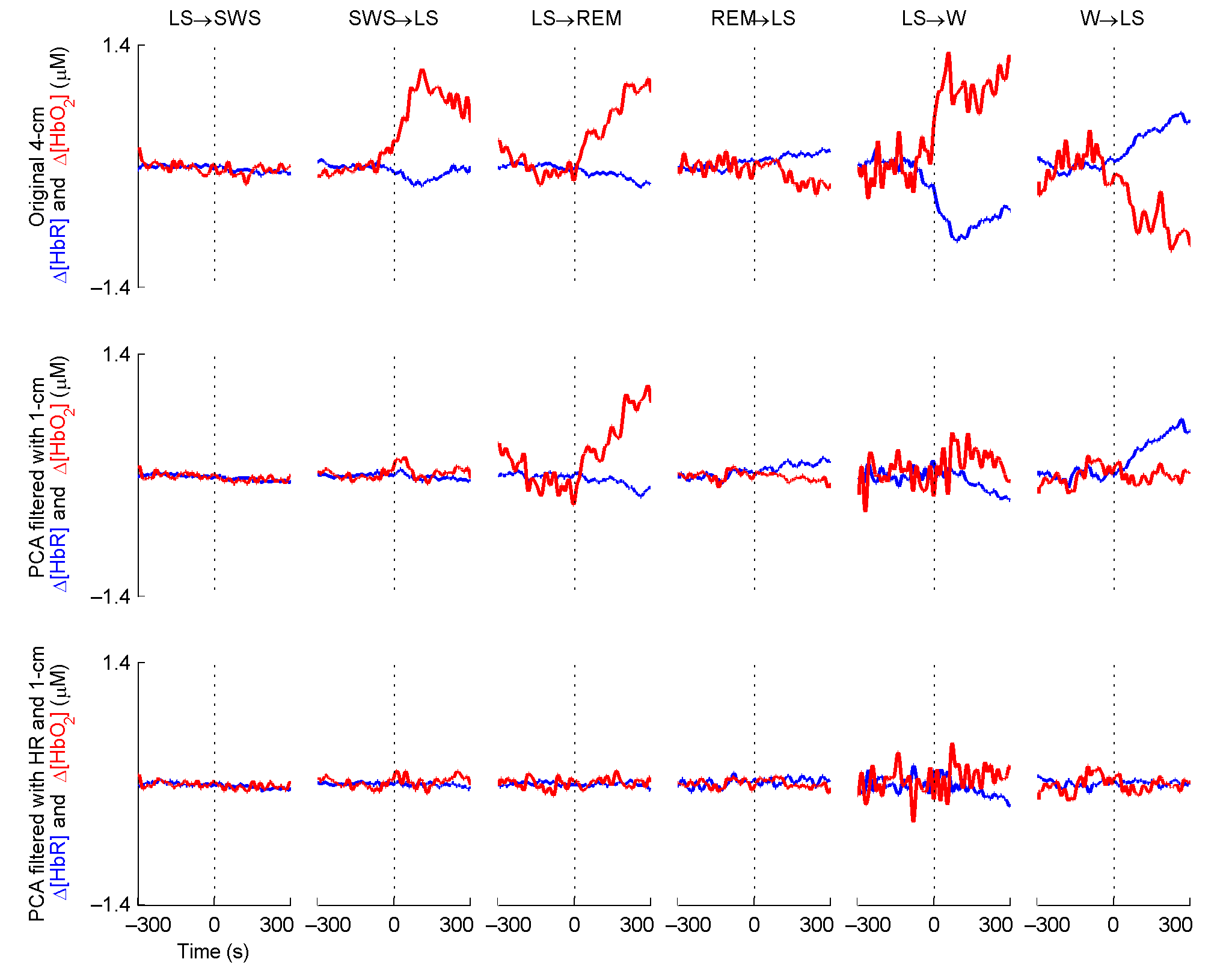

Supplement: Figure S1 — Time course averages around sleep transitions after PCA filtering. The first row shows the original 4-cm transition averages, the second row the 4-cm averages after removing the component corresponding to the 1-cm averages, and the third row the 4-cm averages after removing components corresponding to HR and the 1-cm averages. LFOs have been removed from the signals for graphical purposes. Note that the PCA filtering removes all visible changes from the 4-cm averages, suggesting that any cortical hemodynamic changes either correlate with systemic hemodynamics or are too small to be detected. In some cases, the PCA-filtered averages of the second row still visually resemble the 1-cm averages. This is most likely because the identification and removal of the 1-cm component was based on a simple statistical criterion instead of visual inspection or a complex physiological or anatomical model. (TIF) [file pone.0025415.s001.tif]
